# Air pollution exposure during pregnancy and reduced birth size: a prospective birth cohort study in Valencia, Spain

**DOI:** 10.1186/1476-069X-9-6

**Published:** 2010-01-29

**Authors:** Ferran Ballester, Marisa Estarlich, Carmen Iñiguez, Sabrina Llop, Rosa Ramón, Ana Esplugues, Marina Lacasaña, Marisa Rebagliato

**Affiliations:** 1 Center for Public Health Research (CSISP), Conselleria de Sanitat, Avda Catalunya 21, 46020, Valencia, Spain; 2 Spanish Consortium for Research on Epidemiology and Public Health (CIBERESP), Doctor Aiguader 88, 08003, Barcelona, Spain; 3 School of Nursing, Universitat de València, C Jaume Roig s/n 46010, Valencia, Spain; 4 General Directorate of Public Health. Conselleria de Sanitat, Avda Catalunya 21, 46020, Valencia, Spain; 5 Andalusian School of Public Health (EASP), Campus de la Cartuja s/n, Granada, Spain; 6 Department of Public Health, Rey Juan Carlos University, 28922, Alcorcón, Madrid, Spain

## Abstract

**Background:**

Maternal exposure to air pollution has been related to fetal growth in a number of recent scientific studies. The objective of this study was to assess the association between exposure to air pollution during pregnancy and anthropometric measures at birth in a cohort in Valencia, Spain.

**Methods:**

Seven hundred and eighty-five pregnant women and their singleton newborns participated in the study. Exposure to ambient nitrogen dioxide (NO_2_) was estimated by means of land use regression. NO_2 _spatial estimations were adjusted to correspond to relevant pregnancy periods (whole pregnancy and trimesters) for each woman. Outcome variables were birth weight, length, and head circumference (HC), along with being small for gestational age (SGA). The association between exposure to residential outdoor NO_2 _and outcomes was assessed controlling for potential confounders and examining the shape of the relationship using generalized additive models (GAM).

**Results:**

For continuous anthropometric measures, GAM indicated a change in slope at NO_2 _concentrations of around 40 μg/m^3^. NO_2 _exposure >40 μg/m^3 ^during the first trimester was associated with a change in birth length of -0.27 cm (95% CI: -0.51 to -0.03) and with a change in birth weight of -40.3 grams (-96.3 to 15.6); the same exposure throughout the whole pregnancy was associated with a change in birth HC of -0.17 cm (-0.34 to -0.003). The shape of the relation was seen to be roughly linear for the risk of being SGA. A 10 μg/m^3 ^increase in NO_2 _during the second trimester was associated with being SGA-weight, odds ratio (OR): 1.37 (1.01-1.85). For SGA-length the estimate for the same comparison was OR: 1.42 (0.89-2.25).

**Conclusions:**

Prenatal exposure to traffic-related air pollution may reduce fetal growth. Findings from this study provide further evidence of the need for developing strategies to reduce air pollution in order to prevent risks to fetal health and development.

## Background

In recent years a growing body of epidemiological research has focused on the potential impact of prenatal exposure to air pollution on birth outcomes. Several outcomes have been related to exposure to air pollution during pregnancy, including low birth weight, reduced birth size, and intrauterine growth retardation [1-4]. Moreover, reduction in fetal growth has been associated with poor neurological development as well as with an increased risk for chronic diseases later in life [5,6].

A cohort study is the design of choice for evaluating the impact of air pollution on fetal growth as pregnancy is a process in which the relationship between a given type of exposure and an associated effect may be observed in a limited period of time [7]. Some of the studies carried out on this topic have included large populations using birth data from health care registries [8-10] whereas other cohort studies had smaller samples, but more detailed, primary data [11-13]. Authors of recent methodological reviews [7,14-16] agree that new prospective studies should allow for adequate assessment of air pollution exposure, consider different time windows of exposure, and collect sufficient information on confounding variables.

Nitrogen dioxide (NO_2_) is the air pollutant most frequently used as a surrogate for traffic-related pollution in prospective studies, both in adults and in children [17,18]. This is due to the fact that outdoor NO_2 _levels correlate well with pollutants generated by traffic, they can be easily measured using passive samplers, and they are routinely measured by air quality networks, which allows for correction for seasonality.

The INMA study (Spanish Children's Health and Environment) is a prospective multi-centre pregnancy and birth cohort study that seeks to evaluate the role of the environment on fetal development and children's health in the general population in Spain [19]. The objective of this report is to assess the association between residential exposure to outdoor NO_2 _during pregnancy and anthropometric measures at birth.

## Methods

### Study design and population

The present study was based on data from the INMA cohort in Valencia. Between November 2003 and June 2005, 855 pregnant women attending the prenatal population-based screening program at the reference hospital were included in the study. Thirty-five of these women had a spontaneous abortion or fetal death, 33 withdrew from the study or were lost to follow up, and 787 delivered a live, singleton infant. Exposure to outdoor NO_2 _was assessed for 785 of the 787 mother-child pairs in the study, thus making up the final study population. Deliveries took place between May 2004 and February 2006. The study area covered the home addresses of all participants. Approximately 10% lived in a typically urban zone (city of Valencia), 50% lived in the metropolitan zone, 35% in a semi-urban zone, and the rest in a typically rural zone. The study area covers 1372 km^2 ^including 34 municipalities and has a reference population of almost 300,000 inhabitants with a broad socio-demographic and environmental heterogeneity. The study protocol was approved by the Ethics Committee of the reference hospital and informed consent was obtained from every participating woman. The mothers' recruitment and follow up procedures have been previously reported [19].

### Birth outcome assessment

Outcome variables were birth weight (in grams), birth length and head circumference (in centimetres). Birth weight was measured by the midwife that attended the birth, whereas birth length and head circumference were measured by a nurse when the newborn arrived in the hospital ward within the first twelve hours of life. The three measures were standardized for gestational age and sex using the residuals method [20]. An early ultrasound of the crown-rump length was also available and used for gestational dating when the difference with the last menstrual period was equal to or greater than 7 days. This happened in 11.9% of the cases. We defined small for gestational age (SGA) as a birth weight or length below the 10^th ^percentile according to standard percentile charts for sex and gestational age in the Spanish population [21]. We did not classify SGA in terms of head circumference because our measurement procedure was different from that used in the published charts. Of all the births, 6.4% were classified as preterm births (i.e. gestational age < 37 weeks) in the studied cohort.

### Assessment of air pollution exposure

**Figure 1:**
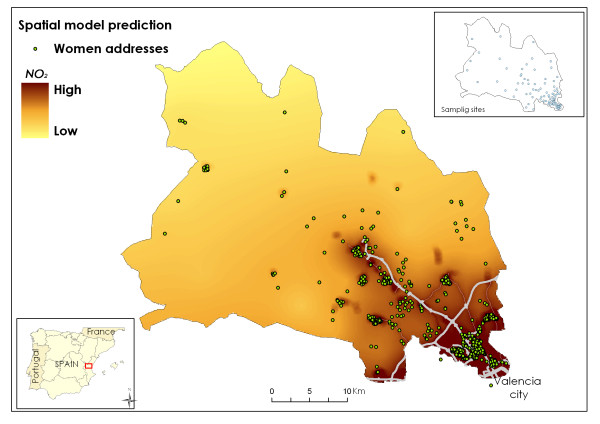
**Spatial distribution of the NO_2 _levels in the study area and addresses of the women in the cohort**.

A procedure was designed to assess individual exposure to NO_2 _as a marker of outdoor air pollution considering both spatial and temporal variations on exposure. Ambient NO_2 _concentrations for 93 sampling points covering the study area were obtained using radial symmetry passive samplers (Radiello^®^, Fondazione Salvatore Maugeri, Padua/Italy) which remained exposed for four sampling periods of 7 days each. The campaigns took place in April, June, and November 2004 and February 2005. The passive samplers were distributed over the area according to geometrical criteria, taking into account the expected pollution gradients and the expected number of births (Figure 1). For obtaining estimates of the NO_2 _spatial distribution in the study area, a two step approach was used. First, universal kriging was used to predict NO_2 _levels at unmonitored sites, i.e. the women's residences. Then, geographical information system (GIS) data (traffic, i.e. vehicle density and distance to a main road, land use, and altitude) were used to improve predictions with the aid of land use regression (LUR).

In addition, in order to take into account temporal variations in exposure, we used daily information from seven stations of the monitoring network within 5 km or less of the study area to adjust NO_2 _spatial estimations to correspond with the pregnancy period for each woman. Thus, the NO_2 _spatial estimation for each woman's residence was multiplied by the ratio between the NO_2 _monitoring network average during the pregnancy period of that particular woman divided by the NO_2 _monitoring network average for the entire study period. In order to explore critical exposure windows, an air pollution exposure indicator for each trimester of pregnancy was constructed using the same procedure as that utilized for the entire pregnancy. Address changes were taken into consideration when they accounted for a relevant fraction of each exposure window (>2/9). The methodology and results for assignment of personal air pollution exposure have been described elsewhere [22].

### Covariates and potential confounders

The mothers completed a detailed questionnaire about socio-demographic characteristics, environmental exposures, and life style variables twice during their pregnancy (weeks 10-13 and 28-32). The questionnaires were administered during personal interviews by previously trained interviewers. Potential confounders included maternal variables (see Additional file 1), infant's sex, paternal height, and season of delivery. Body mass index (BMI) and gestational weight gain were further classified following the Institute of Medicine guidelines [23]. Socio-economic status (SES) was classified using an adaptation of the British SES classification. Environmental tobacco smoke exposure was assessed as both passive exposure at home and global exposure.

### Statistical Methods

We first performed bivariate analysis to determine parental and pregnancy characteristics associated with birth outcomes. We also examined individual NO_2 _levels and maternal and pregnancy characteristics. Association between exposure to residential outdoor NO_2 _and anthropometric measures was assessed by means of linear regression for continuous variables and logistic regression for SGA. In order to avoid excessive influence of extreme values, robust methods were applied. For continuous variables, we checked for the shape of the relation using graphical smoothing techniques. The height of both parents showed a linear relation and was therefore included as a continuous variable in the models. The rest of the continuous variables were categorized to account for non-linear associations. Covariates were retained in the final model if they were related to the outcome based on likelihood ratio (LR) tests with a *p *value of < 0.10 or if they changed effect estimates for the exposure of interest by > = 10% when excluded from the model. The mother's age was included in all models in spite of its statistical significance. Zone of residence was not included in the multivariate analyses because it was highly correlated with NO_2 _levels. To assess the shape of the relationship between measures at birth and NO_2 _levels, we used adjusted GAM models to evaluate the linearity of the relation between NO_2 _levels and the reproductive outcomes, comparing models with NO_2 _levels in a linear and non-linear manner (a cubic smoothing spline with 2, 3, and 4 degrees of freedom) by means of graphical examination and an LR test (p < 0.05).

## Results

Characteristics of the newborns, the mothers and their pregnancy, and the fathers' height in relation to size measures and SGA are described in Additional file 1. In brief, older mothers, mothers who had higher pre-pregnancy weight and/or BMI, who were taller, of higher social class, non-smokers, and of Latin American origin had infants with a higher birth weight and a lower proportion of SGA (in weight) babies. Primiparous mothers, those with low weight gain, those with only primary school education, and those who still smoked at week 12 had infants with a lower birth weight and a higher proportion of SGA (in weight) babies. Boys weighed more than girls. Similar patterns were found for birth length and head circumference adjusted for gestational age, and for SGA (in length) except that there were no differences by country of origin, and in the case of length, no differences by either social class or education were observed. Finally, taller fathers had bigger babies and a lower proportion of SGA babies.

The spatial distribution of NO_2 _levels throughout the study area showed a gradient from the urban zone to the rural one with the two motorways crossing the area playing an important role (Figure 1). The mean residential outdoor NO_2 _level corresponding to the 785 pregnancy periods was 36.9 μg/m^3 ^(Table 1). For 43.2% of the women, the outdoor NO_2 _levels at their residences during the pregnancy period were above 40 μg/m3, the World Health Organization guideline for annual NO_2 _concentration [24]. Individual NO_2 _levels for each trimester correlated well with NO_2 _levels for the whole pregnancy, and moderately between themselves (Table 1).

**Table 1:** Descriptive statistics of the estimates of individual exposure to ambient NO_2 _during the different pregnancy periods.

Pregnancy period	Mean (μg/m^3^)	Percentiles (μg/m^3^)			Pearson's correlation (r) between periods		
		
		25	50	75	First trimester	Second trimester	Third trimester
**First trimester**	37.9	28.2	38.1	48.5			

**Second trimester**	35.9	26.5	35.2	44.2	0.69*		

**Third trimester**	37.0	27.3	37.0	46.1	0.34*	0.65*	

**Whole pregnancy**	36.9	29.4	37.9	45.6	0.80*	0.92*	0.83*

*p < 0.001

INMA-Valencia cohort, 2003-2006

### Air pollution exposure and anthropometric measures

Unadjusted analysis considering the variables in their continuous form showed a negative relationship between individual exposures to ambient NO_2 _and anthropometric measures at birth (Table 2). This relation was statistically significant for first trimester exposure and for both birth length and head circumference, as well as for second trimester exposure and head circumference. After adjustment for covariates and potential confounders, the same temporal pattern persisted (Table 2). Although 95% confidence intervals yielded results that do not reject the null hypothesis, birth head circumference and NO_2 _exposure in the first trimester were marginally associated. Specifically, an increase in 10 μg/m^3 ^in NO_2 _levels during the first trimester of pregnancy was associated with a decrease in head circumference by -0.07 cm (95% CI: -0.14 to 0.005).

**Table 2:** Association between individual exposure to ambient NO_2 _in different time periods during pregnancy and anthropometric measures at birth.*

	Birth weight (in g)^a^ (n:785)				Birth length (in cm)^a^ (n:784)				Birth head circumference (in cm)^a^ (n:782)			
**NO_2 _exposure period**	**β**	**(95% CI)**		**Linearity (df)^b^**	**β**	**(95% CI)**		**Linearity (df)^b^**	**β**	**(95% CI)**		**Linearity (df)^b^**

**Unadjusted**												

First trimester	-3.564	(-23.698;16.570)		L	-0.092	(-0.177;-0.008)		NL (4)	-0.069	(-0.133;-0.004)		L

Second trimester	-4.464	(-25.175;16.248)		NL (3)	-0.050	(-0.137;0.037)		NL (2)	-0.071	(-0.137;-0.004)		L

Third trimester	-5.740	(-26.553;15.072)		L	-0.010	(-0.096;0.077)		NL (4)	-0.017	(-0.084;0.049)		L

Whole pregnancy	-5.792	(-30.065;18.481)		NL (3)	-0.063	(-0.165;0.038)		L	-0.074	(-0.152;0.003)		L

**Adjusted^c^**												

First trimester	-12.782	(-34.537;8.972)		NL (3)	-0.066	(-0.149;0.017)		NL (4)	-0.066	(-0.137;0.005)		L

Second trimester	-9.961	(-32.594;12.671)		NL (4)	-0.040	(-0.125;0.044)		NL (3)	-0.060	(-0.133;0.014)		NL (3)

Third trimester	-4.294	(-25.923;17.335)		L	-0.005	(-0.089;0.079)		NL (2)	-0.028	(-0.099;0.042)		L

Whole pregnancy	-9.729	(-33.218;13.760)		L	-0.047	(-0.146;0.052)		NL(2)	-0.058	(-0.134;0.018)		NL (3)

* Estimates are expressed as the change in birth anthropometric measures for a 10 μg/m^3 ^increase in the mean NO_2 _levels at each woman's residence during the corresponding period. Unadjusted and adjusted models.

^a ^Standardized for gestational age.

^b ^Shape of the relationship after contrast between model with NO_2 _in non-linear vs. linear form; L: linear; NL: non-linear (and degrees of freedom of the selected model).

^c ^Adjusted for:

-Birth weight = maternal age, maternal pre-pregnancy weight, maternal height, paternal height, gestational weight gain, parity, maternal education, smoking during pregnancy, country of origin, sex of the infant, and season of last menstrual period.

-Birth length = maternal age, maternal height, gestational weight gain, parity, maternal education, smoking during pregnancy, working status in the first trimester, country of origin, and sex of the infant.

-Birth head circumference: maternal age, maternal pre-pregnancy weight, maternal height, gestational weight gain, parity, maternal education, smoking during pregnancy, country of origin, sex of the infant, and season of last menstrual period.

**Figure 2:**
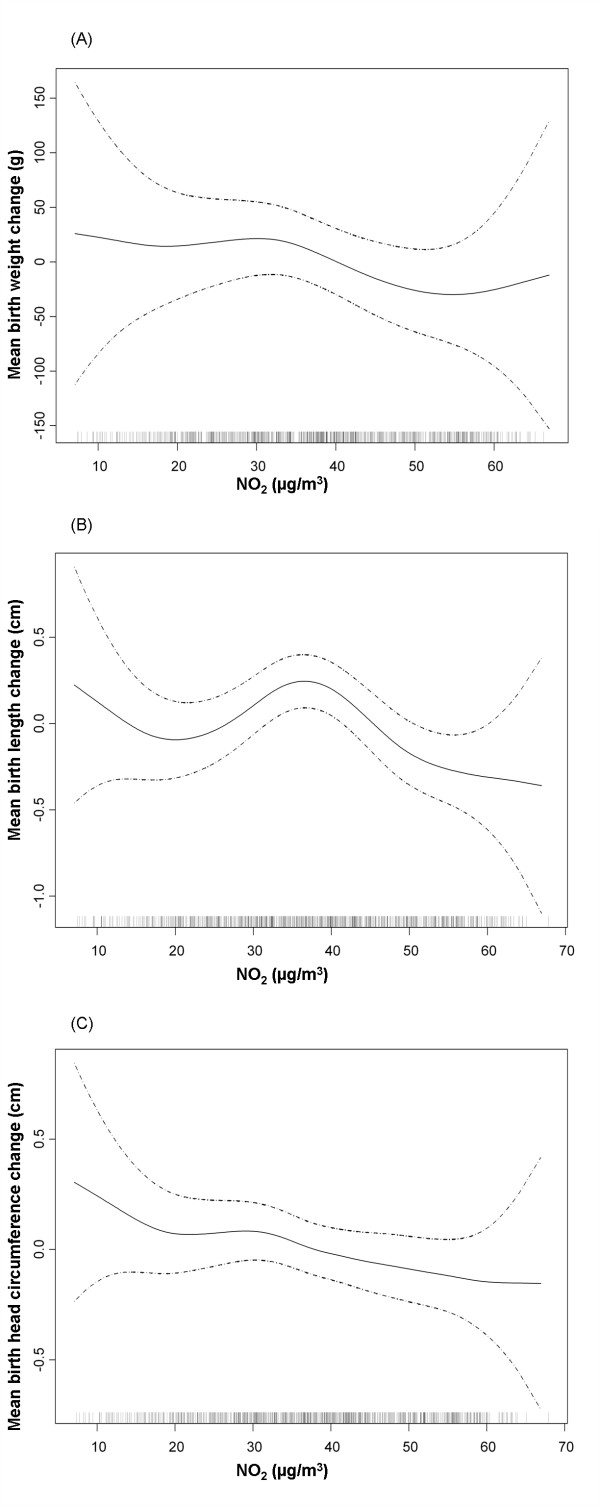
**Relationship between individual NO_2 _exposure during the first trimester and anthropometric measures at birth**. Graphical estimation of the association and 95% confidence intervals for the non-linear model with lower AIC (degrees of freedom: df). (A). Birth weight (gr) and NO_2 _exposure (3 df) B). Birth length (cm) and NO_2 _exposure (4 df). (C). Birth head circumference (cm) and NO_2 _exposure (4 df). *Footnote for Figure 2(C)*: For birth head circumference the model with the best adjustment was the linear model.

When the shape of the relation between NO_2 _exposure and anthropometric measures was assessed, a non-linear relationship was observed. In most cases in the multivariate analysis, the best fit was obtained when NO_2 _was introduced as a cubic smoothing spline with 3 or 4 degrees of freedom (Table 2). Graphic examination of the relation between NO_2 _exposure during the first trimester and birth weight and length, and between NO_2 _exposure during the second trimester and head circumference suggested a change in slope around 40 μg/m^3 ^(Figure 2). For this reason, the association between NO_2 _exposure and weight, length, and head circumference at birth was also analyzed considering NO_2 _as a categorical variable, i.e. >40 μg/m^3 ^versus ≤40 μg/m^3 ^(Table 3). Results of the multivariate analysis indicated that NO_2 _exposure above 40 μg/m^3 ^during the first trimester was associated with a reduction in birth length of -0.27 cm (95%CI -0.51 to -0.03). Birth weight was just marginally associated with NO_2 _exposure; i.e. a reduction of -40.3 grams in birth weight (95%CI: -96.3 to 15.6) for the same comparison. Also a significant reduction in head circumference was found for exposures above 40 μg/m^3 ^throughout the entire pregnancy.

**Table 3:** Association between individual exposure to ambient NO_2 _>40 μg/m^3 ^in different time periods during pregnancy and anthropometric measures at birth.*

	Birth weight (in g)^a^ (n:785)		Birth length (in cm)^a^ (n:784)		Birth head circumference (in cm)^a^ (n:782)	
**NO_2 _exposure period**	**β**	**(95% CI)**	**β**	**(95% CI)**	**β**	**(95% CI)**

**Unadjusted**						

First trimester	-24.309	(-78.256; 29.638)	-0.300	(-0.526; -0.075)	-0.104	(-0.276; 0.069)

Second trimester	-9.648	(-65.156; 45.860)	-0.100	(-0.333; 0.133)	-0.173	(-0.352; 0.005)

Third trimester	28.325	(-26.475; 83.126)	0.150	(-0.079; 0.379)	0.051	(-0.123; 0.226)

Whole pregnancy	-16.912	(-71.233; 37.410)	-0.170	(-0.398; 0.058)	-0.152	(-0.326; 0.022)

**Adjusted^b^**						

First trimester	-40.349	(-96.267; 15.568)	-0.271	(-0.514; -0.028)	-0.074	(-0.257; 0.108)

Second trimester	-37.546	(-96.231; 21.140)	-0.190	(-0.447; 0.066)	-0.177	(-0.368; 0.014)

Third trimester	26.656	(-28.239; 81.551)	0.077	(-0.161; 0.315)	0.011	(-0.167; 0.190)

Whole pregnancy	-33.292	(-84.874; 18.290)	-0.199	(-0.424; 0.027)	-0.171	(-0.339; -0.003)

*Estimates are expressed as the change in birth anthropometric measures comparing NO_2 _exposure levels >40 μg/m^3 ^vs. exposure levels ≤40 μg/m^3 ^at each woman residence during the corresponding period. Unadjusted and adjusted models.

^a ^Standardized for gestational age.

^b ^Adjusted for:

-Birth weight: maternal age, maternal pre-pregnancy weight, maternal height, paternal height, gestational weight gain, parity, maternal education, smoking during pregnancy, country of origin, sex of the infant, and season of last menstrual period.

-Birth length: maternal age, maternal height, gestational weight gain, parity, maternal working status in the first trimester, smoking during pregnancy, country of origin, sex of the infant, and season of last menstrual period.

-Birth head circumference: maternal age, maternal pre-pregnancy weight, maternal height, gestational weight gain, parity, maternal education, smoking during pregnancy, working status in the third trimester, sex of the infant, and season of last menstrual period.

### Analysis of the relationship with small for gestational age (SGA)

**Figure 3:**
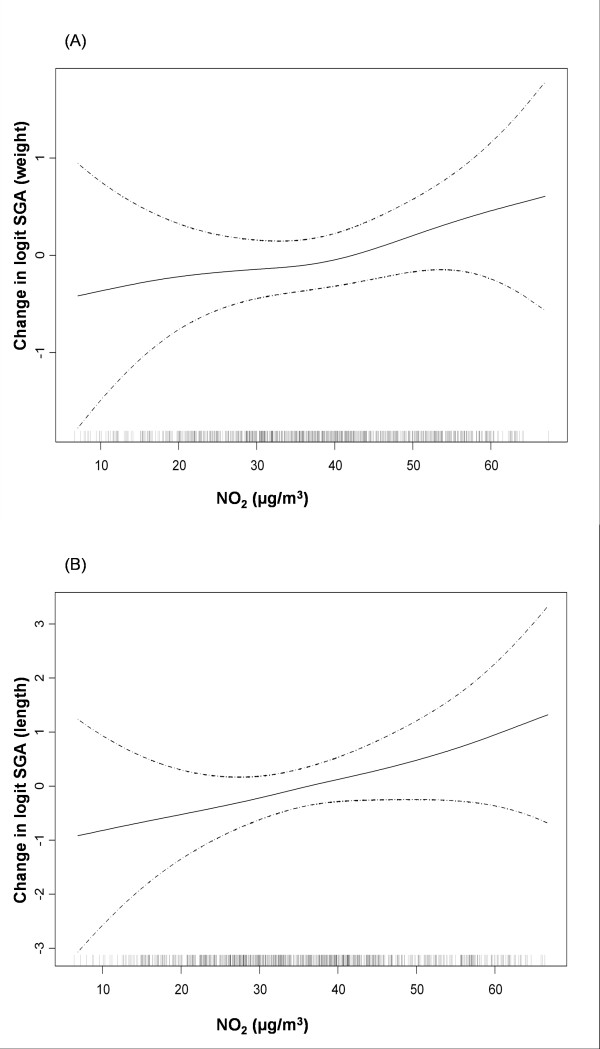
**Relationship between individual NO_2 _exposure during the second trimester and small for gestational age, in birth weight and in birth length in a multivariate analysis**. Graphical estimation of the association and 95% confidence intervals for the non-linear model with lower AIC (degrees of freedom: df). A). Logit of small for gestational age in birth weight and NO_2 _exposure (2 df). *Footnote for Figure 3(A)*: For SGA (in birth weight) the model with the best adjustment was the linear model. (B). Logit of small for gestational age in birth length and NO_2 _exposure (2 df). *Footnote for Figure 3(B)*: For SGA (in birth length) the model with the best adjustment was the linear model.

In the bivariate analysis, although all the odds ratios (OR) were higher than 1, no significant association was found for either of the two measures of SGA and exposure to NO_2 _during pregnancy (Table 4). After adjustment for potential confounders, a clearer association emerged with the second trimester being the most relevant window of exposure. A 10 μg/m^3 ^increase in NO_2 _during the second trimester was thus associated with the risk of SGA-weight, OR: 1.37 (95%CI: 1.01-1.85). For SGA-length the association estimate for the same comparison was OR: 1.42 (95%CI: 0.89-2.25). No significant improvement in the model was obtained with non-linear models for SGA (Figure 3); therefore, we have only included the results for the relationship with NO_2 _exposure as a continuous variable (Table 4).

**Table 4:** Association between individual exposure to ambient NO_2 _in different time periods during pregnancy and Small for Gestational Age (SGA).*

	SGA - weight (n: 785)			SGA - length (n:784)		
**NO_2 _exposure period**	**OR**	**(95% CI)**	**Linearity (df)^a^**	**OR**	**(95% CI)**	**Linearity (df)^a^**

**Unadjusted**						

First trimester	1.013	(0.992; 1.035)	L	1.001	(0.968; 1.035)	L

Second trimester	1.013	(0.992; 1.034)	L	1.006	(0.972; 1.041)	L

Third trimester	1.004	(0.983; 1.026)	L	1.013	(0.979; 1.049)	L

Whole pregnancy	1.014	(0.988; 1.040)	L	1.010	(0.970; 1.052)	L

**Adjusted^b^**						

First trimester	1.182	(0.894; 1.563)	L	1.137	(0.741; 1.744)	L

Second trimester	1.369	(1.013; 1.849)	L	1.416	(0.890; 2.254)	L

Third trimester	1.186	(0.906; 1.552)	L	1.103	(0.750; 1.623)	L

Whole pregnancy	1.281	(0.942; 1.743)	L	1.230	(0.778; 1.945)	L

*Estimates are expressed as the change in odds for SGA (birth weight) and SGA (birth length) for a 10 μg/m^3 ^increase in the mean NO_2 _levels at each woman's residence during the corresponding period. Unadjusted and adjusted models.

^a ^Shape of the relationship after contrast between model with NO_2 _in non-linear vs. linear form; L: linear; NL: non-linear (and degrees of freedom of the selected model).

^b ^Adjusted for:

-SGA in weight: maternal age, maternal pre-pregnancy weight, paternal height, gestational weight gain, parity, maternal education, country of origin, smoking during pregnancy, and season of last menstrual period.

-SGA in length: maternal age, maternal pre-pregnancy weight, maternal education, parity, smoking during pregnancy, gestational weight gain, country of origin, alcohol consumption during pregnancy, and season of last menstrual period.

## Discussion

Results from this mother and child cohort living in a large, heterogeneous area in Valencia, Spain, suggest an association between maternal exposure to outdoor air pollution and birth outcomes. The odds of being SGA-weight increased by 37% when ambient NO_2 _levels increased 10 μg/m^3 ^during the second trimester of pregnancy. For anthropometric measures in continuous form, an association with air pollution appeared for women living in zones with ambient NO_2 _levels above 40 μg/m^3^. The first and second trimesters seem to be the relevant window of exposure.

Results for the different air pollutants varied in the different studies. Besides particulate matter (PM) [either of diameter <10 μm -PM_10_- or <2.5 μm -PM_2.5_-] and carbon monoxide (CO), NO_2 _appears as one of the pollutants more frequently associated with birth outcomes. In a previous review [2] we identified six articles reporting associations between NO_x _or NO_2 _with either birth weight, low birth weight (LBW, measured as birth weight <2500 g), or SGA. The three articles that included nitrogen oxides (NO_x_) were ecological in design and used data from central monitors. None of them found an association between NO_x _and birth weight. For NO_2_, results from the literature reviewed suggested some association with birth weight, but were still not conclusive [8,25,26]. In recent years a considerable number of articles have been published in this field. We have identified 12 articles studying the association of NO_2 _exposure with birth weight that were published after our previous review (Additional file 2) [10,12,27-36]. Of the four studies analyzing birth weight, an association was found in three of them: Bell et al. in Massachusetts and Connecticut (USA) [10], Mannes et al. in Sydney (Australia) [32], and Gouveia and cols in Brazil [29], but no relationship was observed in the Children's Health Study [31]. Interestingly, all but one of the articles [36] studying SGA found an association with NO_2_; in the study in question, however, NO_2 _was the only pollutant studied to be associated with head circumference. As an example, in their study in Vancouver, Brauer et al. [35] estimated residential exposures to air pollution and the risk of SGA. Of the seven air pollutants studied, the association with NO_2 _was the most robust. On the other hand, only three studies found an association between LBW and NO_2 _[10,28,35]. This discrepancy may be due to the fact that the number of cases of SGA is greater than that of LBW term babies, which gives the study more statistical power. Moreover, the use of SGA, calculated for each week of gestation, enables the effect of gestational length to be more effectively controlled than LBW, which is estimated simply by selecting births that take place after a certain period of gestation (i.e. between weeks 37- 44).

Few studies have examined the relation between air pollution exposure during pregnancy and other anthropometric indicators such as birth length or head circumference (HC). Studies of two cohorts of pregnant women in Poland and in New York described a relationship between prenatal exposure to airborne polycyclic aromatic hydrocarbons (PAH) and fetal growth [37]. Regarding prenatal NO_2 _exposure and birth length or HC, a birth register-based study assessed birth length and HC among 26,617 term births in Brisbane, Australia [36]. An IQR range increase in NO_2 _(11.1 μg/m^3^), but not in other pollutants, during the third trimester was associated with a reduction in crown-heel length: -0.15 cm (95%CI: -0.25 to -0.05). Moreover, in the French Eden cohort [38] a reduction of -0.31 cm in HC at birth was found when comparing NO_2 _exposure in the highest tertile (>31.4 μg/m^3^) to that in the lowest tertile. Our results are consistent with the findings of these two studies.

Up to now a clear window of susceptibility for growth retardation has not been identified. In our study we found that exposure during the first trimester is most closely related to a decrease in birth weight and length. In the case of SGA (both, in weight and in length) however, the strongest relationship was found with exposure in the second trimester. Regarding reduced HC, when exposure was evaluated above vs. below 40 μg/m^3^, exposure throughout the pregnancy was the most clearly related. This may indicate that exposure during the entire pregnancy plays the most important role for reduction in the growth of the infant head.

Very few studies have completely assessed the shape of the relationship between air pollution exposure and reproductive outcomes. Instead, most have analyzed the relation using air pollution variables in the continuous form or comparing only two levels. Some have attempted to examine the shape using tertiles or quartiles and observed an increased risk of LBW at higher quartiles [12,28]. Regarding NO_2 _exposure and birth weight, only Ha et al. [25] examined this relationship using GAM models, as did we in the present study. In the former study, although the authors considered the relationship to be relatively linear, a change in the slope may be observed in the figures, with a higher negative gradient after NO_2 _values of around 32 ppb (60 μg/m^3^). In our study, we found some indication of a reduction in birth length starting at a threshold of approximately 40 μg/m^3^. For HC and the risk of SGA we found a monotonic relationship with air pollution exposure.

The biological mechanisms by which air pollutants may affect fetal growth are still unclear. There is some evidence that NO_2 _alters fetal growth and thus may play a causal role. NO_2 _is a potent oxidant and increased lipid peroxidation in the maternal and/or fetal compartment has been found in preterm births [39]. Tabacova et al. investigated the relationship between exposure to nitrogen-oxidizing species and pregnancy complications in an area in Bulgaria highly polluted by oxidized nitrogen compounds [40]. Methemoglobin, a biomarker of individual exposure, was determined, and glutathione balance and lipid peroxide levels were used as measures of oxidant/antioxidant status. A high percentage of women suffered from pregnancy complications, the most common being anaemia (67%), threatened abortion/premature labour (33%), and signs of preeclampsia (23%). Methemoglobin was significantly elevated in all three conditions, in comparison with normal pregnancies. Reduced:total glutathione, an indicator of maternal antioxidant reserves, decreased, whereas cell-damaging lipid peroxide levels increased. More recently, Mohoroviz found similar results for methemoglobin in a polluted area of Croatia [41]. These results suggest that maternal exposure to environmental oxidants can increase the risk of pregnancy complications through stimulation of methemoglobin formation, which may lead to hypoxia and hypoxemia in pregnant women and has an important influence on maternal health as well as on placental and fetal development.

Our study has several limitations. The number of women participating in the study is small compared with that in other studies. Subsequently, the power of the study is fairly low and the estimates have wide confidence intervals. In addition, we had no information available on other important pollutants such as PM_10_, PM_2.5_, sulphur dioxide (SO_2_), and CO, for which some associations with fetal growth have been described in other studies. Consequently, we cannot affirm that NO_2 _is the air pollutant definitively associated with birth measurements. Due to the colinearity between pollutants, NO_2 _may simply be a proxy for other toxins. Still, NO_2 _has been shown to be a marker of air pollution from road traffic [42] and could be a reasonable marker of ultrafine particulates or PAH from this source. Unfortunately, we did not have information on indoor levels of air pollutants. However we did have information on environmental tobacco smoke exposure, an important source of indoor air pollution, and we controlled for this.

Notwithstanding the aforementioned weaknesses, our study has several important strengths. In this prospective study we followed a pregnant cohort from early pregnancy and assessed exposure, health outcomes, and covariates in great detail. In addition, the statistical approach using GAM models allows us to examine the shape of the relationship while the use of robust methods permits the minimization of the influence of extreme values. Moreover, we developed a protocol combining measurements from NO_2 _passive samplers, kriging, and LUR in order to obtain estimates of individual exposure to ambient NO_2 _for each woman. We also performed four different campaigns to assess the stability over time of the spatial NO_2 _distribution in the study area, as recommended by Ritz and Wilhelm [15]. Our method allowed us to address local heterogeneity in order to assign an individual estimate of the exposure, a problem that has been reported to affect other studies [15,30]. Lastly, our study had access to detailed information about each woman's residence throughout pregnancy, including changes of location and address.

## Conclusions

Findings from this mother and birth cohort study in Valencia, Spain, suggest that prenatal exposure to outdoor air pollution, measured as NO_2_, affects the anthropometric development of the fetus, reducing its length and head circumference and increasing the risk of having a small for gestational age (in weight) baby.

We found an association between exposure to levels of NO_2 _above 40 μg/m^3 ^during the first trimester of pregnancy and a reduction in birth weight. This association was only marginal for birth length.

For head circumference (HC) reduction and the risk of SGA, a monotonic relationship with air pollution exposure was observed. The relevant period of exposure for the risk of SGA was the second trimester. Exposure throughout the pregnancy played the most important role in decreased HC.

Compared with other recent studies, NO_2 _levels in the study area occupy an intermediate position; therefore, the results are not due to extreme exposure conditions. Taking into account the relationship between fetal growth reduction and child development and health, strategies should be developed to reduce air pollution in order to prevent these risks.

## Abbreviations

BMI: Body mass index; BSP: Black smoke particles; CI: confidence interval; CO: carbon monoxide; GAM: generalized additive models; GIS: geographical information system; HC: head circumference; INMA: Spanish Children's Health and Environment study; IQR: Interquartile range; LBW: low birth weight (measured as birth weight <2500 g); LR: likelihood ratio; LUR: land use regression; NO_2_: nitrogen dioxide; NO_x_: nitrogen oxides; OR: odds ratio; PAH: polycyclic aromatic hydrocarbons; PM: particulate matter; PM_10_: particulate matter of diameter <10 μm; PM_2.5_: particulate matter of diameter <2.5 μm; ppb: parts per billion; PR: prevalence ratio; SES: socio-economic status; SGA: small for gestational age; SO_2_: sulphur dioxide.

## Competing interests

The authors declare that they have no competing interests.

## Authors' contributions

Authors contributed to the article as follows: FB conceived the study, supervised the data collection and data analysis, and prepared the manuscript. ME contributed to data collection, conducted the data analysis of the association of interest, and helped with manuscript preparation. CI prepared the outcome variables, developed the land use regression analysis, assisted with data analysis, and helped with data interpretation and manuscript preparation. SL, AE, RR, ML, and MR contributed to data collection, provided critical revision of the manuscript, and helped with data interpretation and manuscript preparation. All authors have read and given final approval of the version to be published.

## Supplementary Material

Additional file 1 **Characteristics of pregnant women and their association with birth outcomes in the INMA-Valencia cohort, 2003-2006**. Table with the distribution of the outcome variables among the categories of the covariates at study. Click here for file

Additional file 2 **Results from studies assessing NO_2 _effect on birth weight published between 2003-2008**. Table summarizing the design and main results of studies published between 2003-2008 on air pollution exposure during pregnancy that included NO2 as air pollution indicator and birth weight. Click here for file
