# Supplementary material for: Air pollution exposure during pregnancy and reduced birth size: a prospective birth cohort study in Valencia, Spain
Source: Environ Health. 2010 Jan 29;9:6. doi: 10.1186/1476-069X-9-6 (PMC2845572; doi:10.1186/1476-069X-9-6)
Supplement: Additional file 1 — Characteristics of pregnant women and their association with birth outcomes in the INMA-Valencia cohort, 2003-2006. Table with the distribution of the outcome variables among the categories of the covariates at study. [file 1476-069X-9-6-S1.DOC]

**Additional file 1. Characteristics of pregnant women and their association with birth outcomes in the INMA-Valencia cohort, 2003-2006.**

| **Characteristics** | |  |  | **Birth weight (gram)^b^** | | | **SGA^d^  (weight)** | | **Birth length (cm)^b^** | | | **SGA^d^  (length)** | | **Birth head circumference (cm)^b^** | | |
| --- | --- | --- | --- | --- | --- | --- | --- | --- | --- | --- | --- | --- | --- | --- | --- | --- |
| **Categorical variables** | | **N^a^** | **(%)** | **Mean** | **(SD)** | **p^c^** | **%** | **p^e^** | **Mean** | **(SD)** | **p^c^** | **%** | **p^e^** | **Mean** | **(SD)** | **p^c^** |
| **Maternal age (years)** | <25 | 85 | (11) | 3236.9 | (414.6) | 0.012 | 10.6 | 0.198 | 50.2 | 1.6 | 0.167 | 7.1 | 0.281 | 34.0 | 1.3 | 0.006 |
|  | 25-29 | 274 | (35) | 3301.4 | (417.0) |  | 11.7 |  | 50.5 | 1.9 |  | 6.2 |  | 34.2 | 1.4 |  |
|  | 30-34 | 301 | (38) | 3329.9 | (442.7) |  | 13.7 |  | 50.6 | 2.0 |  | 6.4 |  | 34.4 | 1.3 |  |
|  | >=35 | 125 | (16) | 3424.1 | (435.6) |  | 6.4 |  | 50.7 | 1.7 |  | 2.4 |  | 34.5 | 1.3 |  |
| **Prepregnancy Weight (Kg)** | <50 | 57 | (7) | 3077.2 | (396.5) | <0.001 | 26.3 | 0.006 | 49.9 | 2.1 | <0.001 | 12.3 | 0.014 | 33.7 | 1.2 | 0.001 |
|  | 50-59 | 313 | (40) | 3264.5 | (383.1) |  | 11.9 |  | 50.3 | 1.7 |  | 7.7 |  | 34.2 | 1.3 |  |
|  | 60-69 | 235 | (30) | 3394.6 | (415.6) |  | 8.1 |  | 50.8 | 1.9 |  | 3.0 |  | 34.4 | 1.3 |  |
|  | >=70 | 180 | (23) | 3417.2 | (495.4) |  | 10.6 |  | 50.8 | 1.9 |  | 3.9 |  | 34.5 | 1.3 |  |
| **Gestational weight gain^f^** | Low | 190 | (24) | 3234.1 | (440.6) | 0.001 | 18.0 | 0.011 | 50.3 | 2.0 | 0.045 | 9.5 | 0.053 | 34.2 | 1.3 | 0.357 |
|  | Normal | 286 | (37) | 3320.2 | (390.7) |  | 9.4 |  | 50.6 | 1.8 |  | 4.2 |  | 34.3 | 1.3 |  |
|  | High | 300 | (39) | 3382.5 | (457.3) |  | 9.7 |  | 50.7 | 1.8 |  | 5.0 |  | 34.4 | 1.4 |  |
| **Pre-pregnancy BMI^f^** | <19.8 | 116 | (15) | 3231.2 | (423.0) | 0.010 | 16.4 | 0.202 | 50.3 | 1.8 | 0.187 | 7.8 | 0.563 | 34.1 | 1.3 | 0.099 |
|  | 19.8-26 | 479 | (61) | 3323.3 | (408.5) |  | 10.3 |  | 50.5 | 1.9 |  | 5.7 |  | 34.3 | 1.4 |  |
|  | >26 | 189 | (24) | 3384.9 | (484.6) |  | 11.6 |  | 50.7 | 1.9 |  | 4.8 |  | 34.3 | 1.3 |  |
| **Parity** | 0 | 433 | (55) | 3254.3 | (422.6) | <0.001 | 14.8 | 0.001 | 50.4 | 1.8 | 0.012 | 6.7 | 0.188 | 34.2 | 1.4 | 0.005 |
|  | >=1 | 352 | (45) | 3411.7 | (428.0) |  | 7.4 |  | 50.7 | 1.9 |  | 4.5 |  | 34.5 | 1.2 |  |
| **Education** | Incomplete | 26 | (3) | 3488.9 | (445.4) | 0.102 | 3.8 | 0.477 | 51.0 | 2.0 | 0.540 | 0.0 | 0.201 | 34.3 | 1.1 | 0.005 |
|  | Primary school | 240 | (31) | 3329.9 | (450.3) |  | 12.9 |  | 50.6 | 2.0 |  | 7.1 |  | 34.1 | 1.4 |  |
|  | Secondary school | 334 | (43) | 3293.9 | (427.9) |  | 11.4 |  | 50.5 | 1.7 |  | 4.8 |  | 34.4 | 1.3 |  |
|  | University | 185 | (24) | 3351.3 | (408.4) |  | 10.9 |  | 50.5 | 1.8 |  | 6.6 |  | 34.5 | 1.4 |  |
| **Working status in the first trimester** | Employed | 546 | (70) | 3319.88 | (430.7) | 0.580 | 11.0 | 0.523 | 50.6 | 1.9 | 0.135 | 5.1 | 0.270 | 34.3 | 1.3 | 0.292 |
|  | Unemployed | 238 | (30) | 3338.45 | (435.1) |  | 12.6 |  | 50.4 | 1.8 |  | 7.2 |  | 34.2 | 1.4 |  |
| **Working status in the third trimester** | Employed | 486 | (63) | 3319.05 | (431.9) | 0.644 | 10.9 | 0.448 | 50.6 | 1.9 | 0.459 | 5.1 | 0.317 | 34.4 | 1.3 | 0.104 |
|  | Unemployed | 291 | (37) | 3333.88 | (434.9) |  | 12.7 |  | 50.5 | 1.9 |  | 6.9 |  | 34.2 | 1.4 |  |
| **Socio-economic status** | I+II | 124 | (16) | 3406.2 | (409.3) | 0.034 | 9.8 | 0.782 | 50.7 | 1.7 | 0.326 | 4.9 | 0.684 | 34.6 | 1.4 | 0.013 |
|  | III | 185 | (24) | 3276.4 | (427.6) |  | 11.4 |  | 50.4 | 1.8 |  | 7.0 |  | 34.2 | 1.4 |  |
|  | IV+V (lower) | 476 | (61) | 3322.5 | (437.1) |  | 12.0 |  | 50.6 | 1.9 |  | 5.5 |  | 34.3 | 1.3 |  |
| **Country of origin** | Spain | 693 | (89) | 3311.9 | (431.2) | 0.003 | 12.1 | 0.054 | 50.5 | 1.9 | 0.157 | 5.8 | 0.850 | 34.3 | 1.3 | 0.599 |
|  | Latin American | 60 | (8) | 3496.2 | (396.0) |  | 3.3 |  | 51.0 | 1.8 |  | 6.7 |  | 34.5 | 1.3 |  |
|  | European | 27 | (3) | 3232.7 | (434.8) |  | 14.8 |  | 50.3 | 1.7 |  | 3.7 |  | 34.2 | 1.1 |  |
| **Living with baby's father** | Yes | 763 | (97) | 3328.7 | (430.7) | 0.142 | 11.3 | 0.350 | 50.5 | 1.9 | 0.830 | 5.9 | 0.104 | 34.3 | 1.3 | 0.962 |
|  | No | 22 | (3) | 3191.6 | (464.1) |  | 18.2 |  | 50.5 | 1.9 |  | 0.0 |  | 34.3 | 1.5 |  |
| **Smoking during pregnancy** | No | 459 | (59) | 3365.2 | (417.7) | <0.001 | 9.8 | 0.052 | 50.7 | 1.8 | <0.001 | 4.8 | 0.023 | 34.4 | 1.3 | 0.003 |
|  | Yes, but gave up before week 12 | 126 | (16) | 3374.0 | (448.7) |  | 10.3 |  | 51.0 | 1.9 |  | 3.2 |  | 34.3 | 1.4 |  |
|  | Still smoking at week 12 | 193 | (25) | 3195.9 | (433.3) |  | 16.6 |  | 50.0 | 1.9 |  | 9.8 |  | 34.0 | 1.3 |  |
| **Global passive smoking** | Not exposed | 199 | (26) | 3373.7 | (443.3) | 0.048 | 10.6 | 0.593 | 50.6 | 1.8 | 0.358 | 7.1 | 0.412 | 34.5 | 1.4 | 0.069 |
|  | Exposed | 569 | (74) | 3303.6 | (425.8) |  | 12.0 |  | 50.5 | 1.9 |  | 5.4 |  | 34.3 | 1.3 |  |
| **Passive smoking at home** | Not exposed | 409 | (53) | 3341.8 | (421.6) | 0.248 | 10.3 | 0.263 | 50.6 | 1.9 | 0.622 | 6.4 | 0.482 | 34.4 | 1.4 | 0.040 |
|  | Exposed | 366 | (47) | 3305.8 | (444.6) |  | 12.8 |  | 50.5 | 1.8 |  | 5.2 |  | 34.2 | 1.2 |  |
| **Alcohol consumption (daily mean in g.)** | 0 | 433 | (56) | 3310.7 | (433.4) | 0.264 | 12.0 | 0.582 | 50.5 | 1.9 | 0.595 | 6.0 | 0.928 | 34.2 | 1.2 | 0.030 |
|  | >0 - <1 | 260 | (34) | 3359.8 | (433.5) |  | 10.0 |  | 50.6 | 1.8 |  | 5.4 |  | 34.5 | 1.5 |  |
|  | >=1 | 80 | (10) | 3290.7 | (416.1) |  | 13.8 |  | 50.6 | 1.8 |  | 6.3 |  | 34.2 | 1.3 |  |
| **Infant sex** | Boys | 415 | (53) | 3393.6 | (446.5) | <0.001 | 12.1 | 0.578 | 50.9 | 1.9 | <0.001 | 5.3 | 0.594 | 34.6 | 1.3 | <0.001 |
|  | Girls | 370 | (47) | 3247.8 | (401.8) |  | 10.8 |  | 50.1 | 1.7 |  | 6.2 |  | 34.0 | 1.3 |  |
| **Season of last menstrual period** | Winter | 259 | (33) | 3328.4 | (425.3) | 0.683 | 10.8 | 0.203 | 50.6 | 1.9 | 0.468 | 5.0 | 0.113 | 34.4 | 1.4 | 0.151 |
|  | Spring | 190 | (24) | 3298.4 | (417.6) |  | 11.6 |  | 50.5 | 1.8 |  | 9.0 |  | 34.3 | 1.3 |  |
|  | Summer | 161 | (21) | 3354.5 | (426.5) |  | 8.1 |  | 50.7 | 1.8 |  | 3.1 |  | 34.3 | 1.4 |  |
|  | Fall | 175 | (22) | 3321.2 | (462.6) |  | 15.4 |  | 50.4 | 1.8 |  | 5.7 |  | 34.1 | 1.3 |  |
| **Residence** | Urban | 70 | (9) | 3339.2 | (422.5) | 0.714 | 14.3 | 0.071 | 50.4 | 1.6 | 0.515 | 5.7 | 0.548 | 34.3 | 1.2 | 0.579 |
|  | Metropolitan | 384 | (49) | 3306.6 | (441.7) |  | 12.5 |  | 50.5 | 1.9 |  | 5.5 |  | 34.2 | 1.4 |  |
|  | Semi-urban | 283 | (36) | 3341.8 | (431.1) |  | 11.0 |  | 50.7 | 1.9 |  | 6.8 |  | 34.4 | 1.3 |  |
|  | Rural | 48 | (6) | 3350.5 | (372.9) |  | 2.1 |  | 50.6 | 1.6 |  | 2.1 |  | 34.4 | 1.4 |  |
| **Total** |  | 785 | 100 | 3224.9 | (431.9) |  | 11.5 |  | 50.5 | 1.9 |  | 5.7 |  | 34.3 | 1.3 |  |

| **Continuous variables** |  |  | **β^g^** | **(SE)^g^** | **p^e^** | **OR^h^** | **(95% CI)^h^** | **β^g^** | **(SE)^g^** | **p^e^** | **OR^h^** | **(95% CI)^h^** | **β^g^** | **(SE)^g^** | **p^e^** |
| --- | --- | --- | --- | --- | --- | --- | --- | --- | --- | --- | --- | --- | --- | --- | --- |
| **Maternal height (cm)** |  |  | 11.00 | (2.4) | <0.001 | 0.96 | (0.93,0.99) | 0.04 | (0.01) | <0.001 | 0.94 | (0.89;0.98) | 0.03 | 0.01 | 0.001 |
| **Paternal height (cm)** |  |  | 6.99 | (2.1) | 0.001 | 0.97 | (0.94,1.00) | 0.02 | (0.01) | 0.011 | 0.96 | (0.92;1.00) | 0.01 | 0.01 | 0.065 |

^a^ Number may not sum up 785 in all variables because some values missing. N for birth length: 784. N for birth head circumference: 782.

^b^ Standardized for gestational age.

^c^ p-value from ANOVA in univariate analysis.

^d^ SGA = small for gestational age.

^e^ p-value from chi-square (likelihood ratio test) in univariate analysis.

^f^ According to 1990 Institute of Medicine guidelines (Abrams et al., 2000). BMI = body mass index (Kg/m^2^).

^g^ Beta coefficient (and standard error) from simple linear regression.

^h^ Odds ratio (95% confidence interval).
