# Supplementary material for: Air pollution exposure during pregnancy and reduced birth size: a prospective birth cohort study in Valencia, Spain
Source: Environ Health. 2010 Jan 29;9:6. doi: 10.1186/1476-069X-9-6 (PMC2845572; doi:10.1186/1476-069X-9-6)
Supplement: Additional file 2 — Results from studies assessing NO2 effect on birth weight published between 2003-2008. Table summarizing the design and main results of studies published between 2003-2008 on air pollution exposure during pregnancy that included NO2 as air pollution indicator and birth weight. [file 1476-069X-9-6-S2.DOC]

**Additional file 2. Results from studies assessing NO_2_ effect on birth weight published between 2003-2008**

| **Study** | **Location (time period)** | **Design**  **Nº of births** | **Outcome(s)** | **Exposure** | | | | **Results**  **β (95% CI) grams**  **OR (95% CI)** | **Adjusted for^a^** | **Other outcomes/ air pollutants studied / comments** |
| --- | --- | --- | --- | --- | --- | --- | --- | --- | --- | --- |
|  |  |  |  | **Assessment:**  **-data source;**  **-individual assignment** | **Mean (SD) NO_2_ levels, in µg/m^3^** | **Pregnancy periods examined** | **NO_2_ increase assessed** |  |  |  |
| **Present study** | Valencia; Spain  (2004-2006) | Cohort of pregnant women  785 | Birth weight  SGA | -4 campaigns using passive samplers at 93 sites, monitoring network, and GIS;  -residential prediction using Kriging + LUR temporally adjusted | 36.9 (11.1) | Trimester;  Entire pregnancy | 10 µg/m^3^ (5.3 ppb) | -β for birth weight  1st trimester: -12.8 (-34.5 to 9.0)  Entire pregnancy: -9.7(-33.2 to 13.8)  -OR for SGA  2nd trimester: 1.37 (1.01-1.85)  Entire pregnancy: 1.28 (0.94-1.74) | 1-19 | Association with birth length and birth head circumference  See tables 2-4 for complete results |
| **Brauer et al., 2008 [35]** | Vancouver, Canada  (1999-2002) | Birth register-based study  70249 | SGA  LBW  excluded <37 wg | -Monitoring network and 2 campaigns using passive samplers at 116 sites;  -nearest and IDW area monitors,  LUR temporally adjusted | 32.5 (range:15.3; 53.6) | Month;  Entire pregnancy | 10 µg/m^3^ (5.3 ppb) | Entire pregnancy: NO_2_ IDW  OR for SGA:1.14 (1.09-1.18)  OR for LBW:1.11 (1.01-1.23) | 1,2,7,8,13,18-22 | SGA also associated with CO, SO_2_, and PM_2.5_ but not with O_3_. |
| **Slama et al., 2007 [12]** | Munich, Germany (1998,1999) | Cohort of pregnant women  1016 | BW<3000 g  among births>2500g and >37 <44 weeks | - 2 campaigns with passive samplers at 40 sites and GIS;  -residential prediction using LUR. | 35.8 (P5th:28.3; P95th: 42.5) | Trimester;  Entire pregnancy | 10 µg/m^3^ (5.3 ppb) ,  Quartiles | PR  1st trimester: 0.96 (0.73- 1.20)  2nd trimester: 1.18 (0.95- 1.44)  3rd trimester: 1.13 (0.91- 1.35)  Entire pregnancy: 1.21 (0.86-1.68)  No significant associations by quartiles | 1,3,5-7,13,14,18, 19 | Significant associations with PM_2.5_ and PM_2.5_ absorbance |
| **Bell et al., 2007 [10]** | Massachusetts and Connecticut , USA (1999-2002) | Birth register-based study  358504 | Birth weight  LBW  excluded <37 wg | Average county-level concentration from monitoring networks | 32.7 (9.4) | Trimester;  Entire pregnancy | 9.0 µg/m^3^ (4.8 ppb) | β for birth weight  1st trimester: nr (-9.6 to -8.8)  Entire pregnancy: -8.9(-10.8 to -7.0)  OR for LBW  Entire pregnancy: 1.027 (1.002-1.051)  Associations for other trimesters were less consistent | 1,2,8,13,18,21,23-28 | Exposures to CO, PM_2.5_, and PM_10_ also lowered birth weight.  SGA was also associated with CO, PM_2.5_, PM_10_, and SO_2_. |
| **Hansen et al., 2007 [36]** | Brisbane, Australia  (2000-2003) | Birth register-based study  21432 | SGA | -4 monitoring stations  -average of measurements | 16.5 (7.7) | Trimester ;  Month | IQR: 11.1 µg/m^3^ (5.9 ppb)  Quartiles | No association between NO_2_ and SGA | 1,2,10,18,19,20,23,24,29 | Effect of NO_2_ (IQR:11.1 µg/m^3^) third trimester on crown-heel length :  -0.15cm (95%CI: -0.25 to -0.05).  No effects for PM_10_, BSP, O_3_ |
| **Liu et al., 2007 [33]** | Calgary, Edmonton, and Montreal, Canada  (1985-2000) | Birth register-based study  386202 | SGA among born between wg37-42 | -2, 4, 8 monitoring stations in each city, respectively;  -mean of measurements in the residential area | 45.1 (IQR:32.9; 55.5) | Trimester ;  Month | 37.6 µg/m^3^ (20 ppb) | OR for Trimester  1st: 1.16 (1.09-1.24)  2nd: 1.14 (1.06- 1.21)  3rd: 1.16 (1.09- 1.24) | 1,2,7,18,19,21,30 | Also associated with CO and PM_2.5_.  In multipollutant models only CO showed robustness while effects of NO_2_ and PM_2.5_ were no longer observed.  No effects for SO_2_ and O_3_. |
| **Mannes et al., 2005 [32]** | Sydney, Australia(1998-2000) | Birth register-based study  138056 | Birth weight  SGA | Average of the monitoring stations in the city | 43.6 (13.9) | Trimester;  one month before birth | 1.88 µg/m^3^ (1 ppb) increase among women living <5 km from a monitoring station | β for birth weight  1st trimester: -26.2(-35.4 to -17.0)  2nd trimester: -28.9(-51.0 to -6.8)  3rd trimester: -22.9(-44.6 to -1.2)  Month before pregnancy: -19.7(-27.8 to -11.5)  OR for SGA  1st trimester: 1.06 (0.99- 1.14)  2nd trimester: 1.14 (1.07- 1.21)  3rd trimester: 1.13 (1.05- 1.21)  Month before pregnancy: 1.07 (1.00-1.14) | 1,2,10,13,18,19,20 | Also associated with CO and PM_10_.  In multipollutant models NO_2_ appeared as the most important pollutant. |
| **Salam et al., 2005 [31]** | California, USA  (1975-1987) | Birth register-based study  3901 | Birth weight  SGA <P15  born wg 37-44  LBW born wg 37-44 | Spatial interpolation from the 3 nearest monitoring stations (in <50 km)  When there are available stations in <5 km, the nearest station data are assigned | 67.9 (29.0) | Trimester;  Entire pregnancy | 47 µg/m^3^ (25 ppb) | β for birth weight:  Entire pregnancy: -7.2 g (-34.7 to 20.4)  OR for SGA  1stTrimester: 1.2 (1.0-1.4)  Entire pregnancy: 1.1 (0.9-1.3)  No association for other trimesters or LBW | 1,2,10,12,13,18,27,31 | O3 exposure during 2nd and 3rd trimester and CO exposure during 1^st^ trimester were associated with reduced birth weight. |
| **Wilhelm and Ritz, 2005 [30]** | Los Angeles County, CA, USA  (1994-2000) | Birth register-based study  106483 | LBW at term  Born between 90-320 days; excluded weight <500g or >5000g | 15 monitoring stations for the county-level analysis  11 stations for the address-level analysis | 73.5 (range:38.7- 116.6 | First month;  Trimester;  6 weeks before birth;  Entire pregnancy | nr | Results from one pollutant models for NO2 are not provided.  No association between NO_2_ and LBW after adjusting for CO and/or PM10. | 1,2,7,8,18,19,23,27,32,33 | Association of LBW with CO and PM_10_ but not with O_3_.  Clearer association for women residing 1 mile from a station |
| **Gouveia et al., 2004 [29]** | Sao Paulo, Brasil  (1997) | Birth register-based study  179460 | Birth weight  LBW at term;  excluded <37wg or weight <1000g >5500g | Mean of the hourly maximum from between 4-12 NO_2_ monitoring stations in the city | 117.9 (51.2) | Trimester | 10 µg/m^3^ (5.3 ppb) | β for birth weight  1st trimester: -7.0 g (-14.3 to 0.3)  No association between NO_2_ and LBW | 1,2,7,10,18,23,24 | An association between LBW and CO and PM_2.5_ exposure during 1^st^ trimester was found |
| **Lee et al., 2003 [28]** | Seoul, Korea  (1996-1998) | Birth register-based study  388105 | LBW born between wg37-44 | Daily mean of the 20 monitoring stations in the city | 61.1 (19.2) | Month;  Trimester;  Entire pregnancy | IQR: 27.6 µg/m^3^ (14.7 ppb) | OR for LBW  1st trimester: 1.02 (0.99-1.04)  2nd trimester: 1.03 (1.01-1.06)  3rd trimester: 0.98 (0.96-1.00)  Entire pregnancy: 1.04 (1.00-1.08) | 1,8,18,19,21,25,34 | An association between LBW and CO, PM_10_, and SO_2_ exposure was found except for 3^rd^ trimester for all pollutants, and for 1^st^ trimester for SO_2_. |
| **Liu et al., 2003 [27]** | Vancouver, Canada  (1986-1998) | Birth register-based study  229085 | SGA  born between wg 37-44  LBW  excluded <500g or <22wg | Mean of the monitoring stations in the residential area of each mother | 36.5(P5th:21.6; P95th: 60.0) | First Month;  Last month;  Trimester | 18.8 µg/m^3^ (10 ppb) | OR for SGA  First month: 1.05 (1.01–1.10)  Last month: 0.98 (0.92–1.03)  1st trimester: 1.03 (0.98–1.10)  2nd trimester: 0.94 (0.88–1.00)  3rd trimester: 0.98 (0.92–1.06)  OR for LBW  First month: 0.98 (0.90–1.07)  Last month: 0.94 (0.85–1.04) | 1,2,7,18,19 | An association between SGA and CO and SO_2_ first month exposure was also found |

Abbreviations: β (95% CI): regression coefficient for birth weight (in grams) and 95% confidence interval; OR: odds ratio; PR: prevalence ratio; SGA: small for gestational age: <10^th^ percentile from population charts unless otherwise indicated in the table; LBW: birth weight <2500gr unless otherwise indicated in the table. LBW at term: LBW among those born ≥37 weeks of gestation; wg: weeks of gestation; IDW: inverse-distance weighting; GIS: geographical information system; LUR: land use regression; nr: not reported; BSP: black smoke particles; ppb: parts per billion; P5th: Percentile 5^th^. P95th: Percentile 95^th^ ; IQR: Interquartile range.

^a^ Covariates considered: 1: Gestational age; 2: Maternal age; 3: Maternal pre-pregnancy weight; 4: Gestational weight gain; 5: Maternal height; 6: Maternal body mass index; 7: Parity; 8: Maternal education; 9: Maternal working status; 10: Maternal socio-economic status; 11: Mother's country of origin; 12:Living with partner; 13: Maternal smoking during pregnancy; 14: Maternal environmental tobacco exposure at home; 15: Maternal environmental general tobacco exposure; 16: Maternal alcohol consumption; 17: Paternal height; 18: Infant sex; 19: Season; 20: Country of origin; 21: Year of birth; 22: Income; 23: Prenatal care; 24: Type of delivery; 25: Birth order; 26: Weather; 27: Mother's ethnicity; 28: Marital status; 29: Previous abortions; 30: Location of residence; 31: Diabetes; 32:Time since last delivery; 33: Previous preterm; 34: Paternal education.
